# Efficacy, safety, and completion of modified short-course rifapentine and isoniazid for latent tuberculosis infection in patients with high-risk rheumatic disease: a multicentre, open-label, randomized, non-inferiority trial

**DOI:** 10.1016/j.eclinm.2026.103831

**Published:** 2026-03-13

**Authors:** Lifan Zhang, Yujie He, Wenwen Wang, Lijun Wu, Xiaoxia Zuo, Sheng Chen, Yanping Zhao, Shengyun Liu, Huaxiang Wu, Cainan Luo, Yaou Zhou, Ran Wang, Juan Zhang, Chunlei Li, Kui Zhang, Wenjie Zheng, Lidan Zhao, Jinjing Liu, Wen Zhang, Baotong Zhou, Guiren Ruan, Qian Wang, Mengtao Li, Yan Zhao, Xiaofeng Zeng, Fengchun Zhang, Yuelun Zhang, Ping Zhu, Hongbin Li, Xiaoqing Liu

**Affiliations:** a Division of Infectious Diseases, Department of Internal Medicine, State Key Laboratory of Complex Severe and Rare Disease, Peking Union Medical College Hospital, Chinese Academy of Medical Sciences & Peking Union Medical College, Beijing, China; b Clinical Epidemiology Unit, Peking Union Medical College, International Clinical Epidemiology Network, Beijing, China; c Center for Tuberculosis Research, Chinese Academy of Medical Sciences and Peking Union Medical College, Beijing, China; d Department of Rheumatology and Immunology, The First Affiliated Hospital of Zhengzhou University, Zhengzhou, China; e Department of Rheumatology, The Second Affiliated Hospital of Zhejiang University School of Medicine, Hangzhou, China; f Department of Rheumatology, People's Hospital of Xinjiang Uygur Autonomous Region, Urumchi, China; g Department of Rheumatology and Immunology, Xiangya Hospital, Central South University, Changsha, China; h Department of Rheumatology, Renji Hospital, School of Medicine, Shanghai Jiao Tong University, Shanghai, China; i Department of Rheumatology and Immunology, The First Affiliated Hospital of Harbin Medical University, Harbin, China; j Department of Rheumatology and Immunology, The Affiliated Hospital of Inner Mongolia Medical University, Hohhot, China; k Department of Clinical Immunology, Xijing Hospital, Fourth Military Medical University, Xi'an, China; l Department of Rheumatology and Clinical Immunology, Peking Union Medical College Hospital, Chinese Academy of Medical Sciences & Peking Union Medical College, National Clinical Research Center for Dermatologic and Immunologic Diseases, Ministry of Science & Technology, Key Laboratory of Rheumatology and Clinical Immunology, Ministry of Education, Beijing, China; m Center for Prevention and Early Intervention, National Infrastructures for Translational Medicine, Institute of Clinical Medicine, Peking Union Medical College Hospital, Chinese Academy of Medical Sciences and Peking Union Medical College, Beijing, China

**Keywords:** Tuberculosis preventive treatment, Rheumatic disease, Efficacy, Safety, Randomized controlled trial

## Abstract

**Background:**

Patients with rheumatic diseases are at high risk for latent tuberculosis infection (LTBI) reactivation. We aimed to evaluate whether a modified 3-month regimen (3HP-PUMCH) was non-inferior to the standard 9-month isoniazid regimen (9H) for tuberculosis preventive treatment in this vulnerable population.

**Methods:**

We conducted a multicenter, open-label, randomized, non-inferiority trial at nine tertiary general hospitals in China. Eligible participants were adults (18–70 years) with high-risk rheumatic diseases and LTBI undergoing immunosuppressive therapy. Patients were randomized (1:1) to receive either the 3HP-PUMCH regimen (twice-weekly rifapentine 450 mg plus daily isoniazid 300 mg) or the 9H regimen (daily isoniazid 300 mg). The primary endpoint was the occurrence of tuberculosis, with a non-inferiority margin of 1.4 percentage points. Analysis used the modified intention-to-treat population. The trial was registered with Chinese Clinical Trial Registry ChiCTR1800018242.

**Findings:**

Between 19 September 2018 and 18 August 2021, 536 patients with rheumatic diseases were enrolled. The cumulative rate of tuberculosis was 0.00% (0 of 249, 95% CI 0.00–1.47) in the 3HP-PUMCH group, compared with 1.15% (3 of 260, 95% CI 0.24–3.34) in the 9H group, with a rate difference of −1.15 percentage points (95% CI −2.4 to 0.14). Drug discontinuation rates due to serious adverse events or tuberculosis occurrence were 2.8% (7 of 249) in the 3HP-PUMCH group and 1.9% (5 of 260) in the 9H group (p = 0.509). Adverse drug reactions occurred in 24 (9.6%) of 249 patients versus 39 (15.0%) of 260 (p = 0.066), with hepatotoxicity in 11 (4.4%) of 249 versus 27 (10.4%) of 260 (p = 0.010). 223 (89.6%) of 249 patients completed treatment in the 3HP-PUMCH and 237 (91.2%) of 260 in the 9H group (p = 0.54).

**Interpretation:**

The short-course 3HP-PUMCH regimen was non-inferior to the 9H regimen in preventing tuberculosis and demonstrated a favorable safety profile, with high treatment completion in LTBI patients with rheumatic diseases. This regimen might be more suitable for patients with underlying diseases and those on concomitant medications.

**Funding:**

10.13039/501100001809National Natural Science Foundation of China.

**Research in context**

**Evidence before this study**

We conducted a comprehensive systematic review and meta-analysis of tuberculosis preventive treatment in patients with rheumatic diseases, which was published in this journal (https://doi.org/10.1016/j.eclinm.2025.103177). Our search of major databases up to January 2025 revealed that, although preventive treatment generally reduced the risk of tuberculosis in rheumatic diseases patients, the evidence base was dominated by heterogeneous cohort studies. A follow-up searching up to November 3, 2025, confirmed that high-level evidence, particularly randomized controlled trials (RCTs), remains extremely limited globally. Furthermore, clinical data on modern, shorter preventive treatment regimens—such as 3-month rifapentine plus isoniazid (3HP)—were virtually absent for this vulnerable group. The lack of RCTs constitutes the largest single gap in current global guidelines for rheumatic diseases patients.

**Added value of this study**

This study directly addresses the critical gap in evidence identified in our prior work. This multi-center randomized non-inferiority trial is one of the first to compare a modified 3HP preventive treatment regimen (3HP-PUMCH) against the long-established 9H regimen in rheumatic diseases patients with latent tuberculosis infection (LTBI). We demonstrate that the 3HP-PUMCH regimen is non-inferior to 9H in preventing tuberculosis. The key added value lies in establishing high-level (RCT) evidence for a viable alternative. The modified regimen was designed to mitigate potential risks in this high-risk population, particularly those with fragile liver/kidney function and complex co-medications. Our 3HP-PUMCH regimen showed significantly lower rates of hepatotoxicity and fewer adverse reactions compared to 9H, with comparable treatment completion rates.

**Implications of all the available evidence**

Our findings offer important initial data that may influence clinical practice and policy discussions. This short-course preventive treatment option presents a promising alternative to the standard 9H regimen, reducing the treatment burden and drug toxicity, which are significant barriers to successful tuberculosis prevention in this population. While acknowledging the limitations in sample size and event rate, our findings represent high-level evidence that supports considering short-course tuberculosis preventive treatment for rheumatic diseases patients. Further large-scale trials are needed to confirm these results across diverse settings and inform updates to international guidelines, ultimately improving tuberculosis preventive treatment strategies for rheumatic diseases patients worldwide.

## Introduction

Tuberculosis and rheumatic diseases are both major global health challenges. China remains a high-burden country, ranking third worldwide for tuberculosis.^1^ An estimated 300 million individuals carry latent tuberculosis infection (LTBI), representing a substantial reservoir for reactivation and transmission.^2^ Implementing tuberculosis preventive treatment (TPT) on such a massive scale is extremely challenging, making the focused management of high-risk populations essential.

Patients with rheumatic diseases are particularly vulnerable to tuberculosis infection and disease.^3^ Rheumatic diseases affect over 20 million people in China, accounting for nearly one-fifth of the global burden. Our previous multicenter investigation reported a tuberculosis prevalence of 882 per 100,000 among patients with rheumatic diseases.^3^ Cohort data indicate that patients with rheumatic disease have an approximately 20-fold higher incidence of tuberculosis than the general population.^4^ This elevated risk likely reflects both immune dysregulation and the frequent use of immunosuppressive therapies. More seriously, once tuberculosis occurs, balancing ongoing immunosuppression against anti-tuberculosis therapy poses a therapeutic dilemma. Immunosuppression increases the risk of dissemination, yielding a higher proportion of extrapulmonary and severe tuberculosis among rheumatic disease patients, with reported fatality rates up to 50%.^5^^,^^6^ The combination of high incidence and high mortality makes tuberculosis prevention and management in rheumatic disease patients a severe challenge for both clinical practice and public health.

The standard WHO-recommended 9-month isoniazid monotherapy (9H) often suffers from poor adherence due to its long duration.^7^ In 2015, WHO recommended a 3-month regimen of weekly rifapentine and isoniazid (3HP) as alternatives.^8^ This regimen has shown favorable efficacy and safety in patients with tuberculosis, children, adolescents, and patients with HIV.^9^, ^10^, ^11^ However, the safety profile of 3HP regimen in the Chinese population has raised concerns. A previous report indicated that more than 70% of patients with silicosis had adverse events when receiving the 3HP regimen, and approximately 30% of them discontinued treatment owing to adverse drug reactions.^12^ Another clinical trial was prematurely terminated in older patients receiving the 3HP regimen because of an unexpectedly high frequency of adverse drug reactions. Furthermore, concomitant medication was identified as an independent risk factor for adverse drug reactions in TPT.^13^ Pharmacokinetic studies have shown that isoniazid is metabolized rapidly, whereas rifapentine is metabolized slowly, in the Chinese population, which may contribute to the high rate of adverse drug reactions.^14^

High-quality randomized controlled trials (RCTs) of TPT in patients with rheumatic diseases are scarce, because clinical heterogeneity, polypharmacy, and the demands of safety oversight make such trials challenging to design and conduct. Implementing the standard weekly 3HP regimen poses additional challenges in this population. Given that many patients with rheumatic diseases have pre-existing organ dysfunction and require multiple immunosuppressive and other concomitant medications, the risk of severe adverse drug reactions observed in previous Chinese trials was judged clinically and ethically unacceptable by the clinical investigators and the institutional ethics committee.

Rather than including the standard 3HP regimen as a comparator, we developed a modified regimen aiming to improve tolerability while maintaining the same weekly rifapentine amount. Specifically, we adopted a dose–frequency modification by splitting rifapentine into two administrations per week (450 mg twice weekly; 900 mg/week) and paired it with daily isoniazid (maximum 300 mg/day) for 3 months. This modified regimen was termed 3HP-PUMCH (Peking Union Medical College Hospital [PUMCH]). Twice-weekly rifapentine dosing has been described in Chinese guidance documents (2015 edition)^15^ and subsequently in the national technical specifications for tuberculosis prevention and control (2020 edition)^16^ in selected clinical scenarios, supporting the feasibility of a twice-weekly schedule in Chinese practice. This 3HP-PUMCH regimen was evaluated in comparison to the standard 9H regimen in high-risk patients with rheumatic diseases and LTBI.

## Methods

### Study design and participants

This multicentre, open-label, non-inferiority RCT was conducted at nine tertiary general hospitals within the ETHERTB collaboration network, representing Eastern, Central, and Western China. ETHERTB, initiated in 2014 by PUMCH, is a multidisciplinary collaboration composed of experts in rheumatology, infectious diseases, tuberculosis, respiratory medicine, and clinical pharmacology. The group focuses on tuberculosis in rheumatic diseases and has conducted nationwide epidemiological investigations and peer-reviewed studies,^3^^,^^17^, ^18^, ^19^, ^20^ which provided the scientific and operational foundation for the present trial. This trial was registered with the Chinese Clinical Trial Registry (ChiCTR1800018242, https://www.chictr.org.cn, registered on September 6, 2018).

Consecutive outpatients and inpatients at the nine participating centers were screened and enrolled between September 2018 and August 2021. Patients were eligible if they were aged 18–70 years; diagnosed with at least one of the high-risk rheumatic diseases, including systemic lupus erythematosus (SLE), Takayasu's arteritis, Behcet's disease (BD), primary Sjogren's syndrome, rheumatoid arthritis (RA) and ankylosing spondylitis; not currently using biologics; taking at least one of the following medications: ≥15 mg daily of prednisone or equivalent, methotrexate, cyclophosphamide, mycophenolate mofetil, cyclosporine A, azathioprine, tacrolimus, leflunomide, iguratimod, Tripterygium wilfordii, and TNF inhibitors; and had a positive T-SPOT.TB test (≥24 SFCs/10ˆ6 PBMC). Patients were excluded if they had suspected or confirmed tuberculosis; seropositive status for HIV-Ab; seropositive status for hepatitis C virus-Ab or hepatitis B virus surface antigen; severe liver damage (total bilirubin >3 mg/dL or aminotransferase level >2 times the upper limit of normal) or liver cirrhosis; previous use of TPT; history of allergy to isoniazid or rifapentine; and pregnancy or lactation. Pregnant or breastfeeding women were excluded due to limited safety data for rifapentine-based preventive therapy in these populations at the time of protocol approval and local ethics requirements.

### Ethics

This trial received ethical approval from the institutional review boards of Peking Union Medical College Hospital (approval No. JS-1498) and all other participating centers. All participants provided written informed consent prior to enrollment.

### Randomization and masking

Participants were randomly assigned in a 1:1 ratio to the 9H and 3HP-PUMCH groups using a computer-generated random sequence. Randomization was stratified by the research center. A random sequence was generated using a fixed-block randomization method with a block length of 4. Allocation concealment was maintained using a web-based central randomization system. This study was an open-label trial, and the enrolled patients and clinicians who administered the drug interventions were aware of the patients’ group assignments. However, to minimize assessment bias, the adjudication of the primary endpoint was performed by a blinded external committee.

### Procedures

The intervention group received the 3HP-PUMCH regimen consisting of isoniazid once daily, 5 mg/kg (with a maximum daily dose of 300 mg, up to 2100 mg/week), combined with twice-weekly rifapentine at 450 mg per dose (900 mg/week), for 3 months. The control group received the 9H regimen, isoniazid once daily, 5 mg/kg (with a maximum daily dose of 300 mg), for 9 months.

If tuberculosis occurred during the study, patients were referred to a tuberculosis specialist for standard anti-tuberculosis treatment. The treatment of baseline rheumatic diseases was conducted following the best available clinical guidelines or consensus at the time of treatment. Subsequently, it was adjusted as needed based on the patient's condition. A uniform treatment regimen for patients with rheumatic diseases was not stipulated in this study.

All enrolled participants were screened for LTBI using the T-SPOT.TB test before randomization. Tuberculosis was excluded prior to randomization using prespecified symptom screening and chest CT. Per the prespecified exclusion criteria, participants with suspected or confirmed tuberculosis were excluded and referred for further evaluation as needed. Data were collected by researchers who received standardized training and used a unified case report form. At baseline, general demographic characteristics, diagnosis and course of rheumatic diseases, use of medications (eg, glucocorticoids or immunosuppressants), and a series of laboratory tests (including hematological parameters, liver and renal function, inflammatory markers, and fasting blood glucose) were collected.

Face-to-face interviews were conducted during the TPT intervention at 1 month, 2 months, and 3 months in the 3HP-PUMCH group. By contrast, interviews were conducted at 1 month, 2 months, 3 months, 6 months, and 9 months in the 9H group. At each visit, clinical assessments and laboratory tests (including hematological parameters and liver and renal function tests) were performed. Medication adherence and adverse events were documented. After completing TPT, patients in both groups were followed up via face-to-face or telephone interviews at 6 months, 12 months, 18 months, and 24 months to assess the occurrence of tuberculosis. Tuberculosis disease status was confirmed for all participants through clinic visits, electronic medical records, or telephone follow-ups. During the COVID-19 pandemic, some scheduled hospital visits for laboratory tests were disrupted; however, clinical adverse events were captured through telephone interviews to ensure patient safety.

The diagnosis of tuberculosis was based on either microbiological confirmation or clinical diagnosis. Microbiologically confirmed tuberculosis was defined when biological specimens (e.g., sputum, blood, or other clinical samples) tested positive by smear microscopy, culture, Xpert MTB/RIF, or other molecular diagnostic techniques. Clinically diagnosed tuberculosis was defined when patients presented with characteristic symptoms (fever, cough, chest pain, night sweats, weight loss) supported by compatible laboratory and imaging findings, together with a positive response to empiric tuberculosis treatment.

All suspected cases of tuberculosis were reviewed by an external Endpoint Event Adjudication Committee, comprising two tuberculosis experts and one rheumatology expert, all blinded to the TPT regimen allocation. The final diagnosis was reached by consensus. The severity of adverse events was assessed using the Common Terminology Criteria for Adverse Events, with grade ≥3 events classified as serious adverse events. The causality of adverse events related to preventive drugs was determined according to the WHO Uppsala Monitoring Centre criteria. These assessments were performed by local clinicians at each subcenter, following standardized guidelines. To ensure consistency across sites, all assessments were periodically reviewed by the study's safety committee, with any discrepancies resolved by expert consensus. The intended treatment duration was 3 months for 3HP-PUMCH and 9 months for 9H. Treatment completion was defined as the intake of at least 22 doses of rifapentine and 81 doses of isoniazid within 16 weeks for the 3HP-PUMCH group, and at least 243 doses of isoniazid within 52 weeks for the 9H group.

### Outcomes

The primary endpoint of this study was the occurrence of tuberculosis during the follow-up period after randomization. Secondary endpoints included permanent discontinuation of TPT due to serious adverse events or the occurrence of tuberculosis, adverse drug reactions related to prophylactic drugs, and completion rate of TPT.

### Statistics

We assumed that patients with six types of high-risk rheumatic diseases, treated with biological agents, immunosuppressive agents, or glucocorticoids (equivalent to prednisone ≥15 mg/day), and diagnosed with LTBI, would receive the 9H regimen, with an annual incidence rate of 1.8% and a 2-year incidence rate of approximately 3.6%. This estimate is based on cohort studies conducted in high tuberculosis burden countries/regions.^21^ The clinically acceptable non-inferiority absolute rate difference between the 3HP-PUMCH and 9H regimens was set at 1.4 percentage points. We hypothesized that the protective effect of the 3HP-PUMCH regimen, which contains rifapentine, would be superior to that of isoniazid monotherapy. The expected rate difference was estimated to be −2.5 percentage points, assuming an incidence rate of 1.1% in the 3HP-PUMCH group. This hypothesis was informed by similar studies in the HIV population, which observed 1.01% vs 3.50% cumulative tuberculosis incidence rates, yielding a risk difference of −2.49 percentage points in favor of 3HP regimen.^11^ To achieve 80% statistical power for a non-inferiority conclusion, a sample size of 236 participants per group was calculated with a 1:1 allocation ratio and a one-sided alpha level of 0.025. Assuming a 10% loss to follow-up, a total of 526 eligible participants were planned for enrollment.

A modified intention-to-treat (mITT) method was used for the primary analysis. The final mITT analysis set included participants who were randomized and received at least one dose of the intervention drug. The demographic and clinical characteristics of the study participants were analyzed using descriptive statistical methods. The primary endpoint was determined using Kaplan–Meier curves. For time-to-event analyses, participants who did not complete the full follow-up period were censored at the date of their last contact. Analyses were conducted using a complete-case approach. The cumulative incidence rate and its 95% CI were estimated using the exact method. We also performed a log-rank test for the primary endpoint. However, the result was interpreted with caution as the test may be underpowered and its asymptotic assumptions may not hold given the infrequent events. A multivariable Cox regression model adjusting for center was not utilized, as sparse events across centers could lead to overfitting and unstable or non-convergent estimates. The difference in cumulative incidence rates between groups and the two-sided 95% CI were calculated. Non-inferiority was concluded if the upper limit of the 95% CI of the cumulative incidence rate difference was less than the pre-specified non-inferiority margin of 1.4 percentage points. In a case where the upper limit of the CI was less than 1.4 percentage points, it was considered that a non-inferiority conclusion was reached. In a case where a non-inferiority result was achieved, the result of superiority was further evaluated as a secondary exploratory analysis by comparing the upper limit of the CI for a cumulative incidence rate difference of 0.0 percentage points.

In addition to the mITT analysis, an ITT analysis (including 17 eligible patients who declined TPT after randomization) was conducted as a sensitivity analysis. A per-protocol (PP) analysis was also performed, considering the non-inferiority design of the study. The PP set included all mITT participants who completed TPT. The ITT and PP analyses followed a strategy similar to the mITT analysis, with the primary outcome analyzed as a sensitivity analysis. In these sensitivity analyses, the incidence density based on the person-years of follow-up was also calculated, and the differences in incidence density and their 95% CI were estimated. Data analysis was performed using the R software (version 4.4.1, www.r-project.org, R Foundation, Vienna, Austria).

### Role of the funding source

The funders of the study had no role in study design, data collection, data analysis, data interpretation, or writing of the report.

## Results

**Fig. 1:**
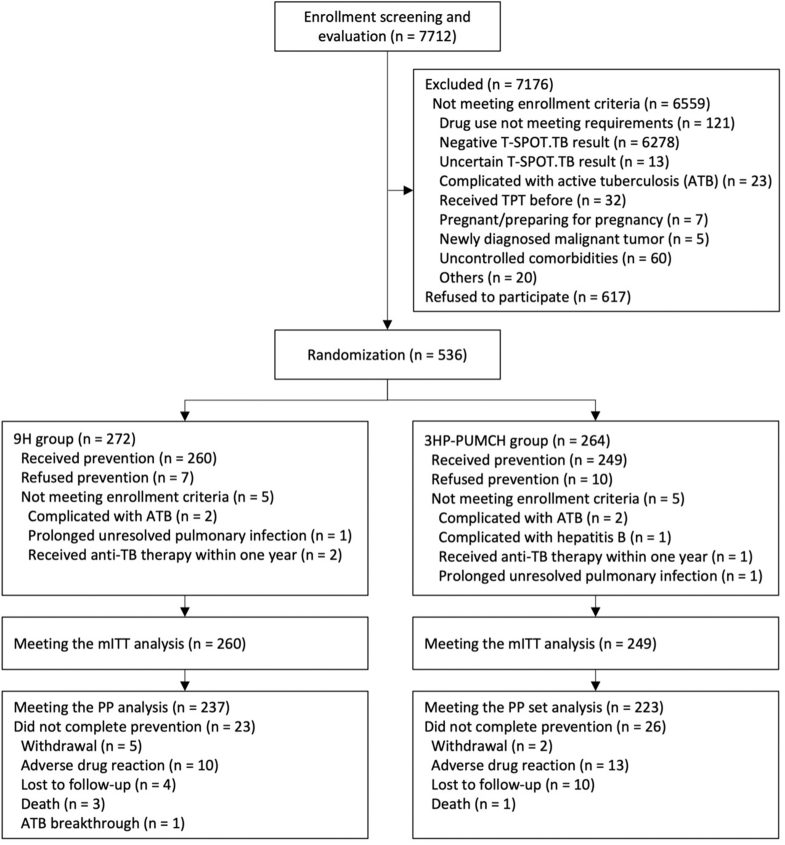
Flowchart of study participant intervention and follow-up.

Between 19 September 2018 and 18 August 2021, 7712 patients with rheumatic diseases were screened for eligibility. Of these, 536 were randomized. However, 17 patients declined to receive anti-tuberculosis drugs, and 10 were found to be ineligible for enrollment. Ultimately, 509 patients who received the TPT regimen were included in the mITT analysis, with 260 patients in the 9H group and 249 patients in the 3HP-PUMCH group. Subsequently, after excluding cases of early drug discontinuation due to adverse events, withdrawal of consent, loss to follow-up, death, and tuberculosis breakthrough, the PP population consisted of 237 patients in the 9H group and 223 in the 3HP-PUMCH group (Fig. 1).

The baseline patient characteristics were comparable between the two study groups. 178 patients (33.2%) were men, and 481 (89.7%) were of Han ethnicity among the 536 patients randomized. The mean patient age was 44 years (SD 13). SLE, RA, and BD were the most prevalent rheumatic diseases, accounting for 70.3% (377/536) of the study participants. The median duration of participation in the study was 27 months for the 3HP-PUMCH group and 33 months for the 9H group (Table 1).

**Table 1:** Baseline patient characteristics.

	3HP-PUMCH group (n = 264)	9H group (n = 272)	All patients (n = 536)
Gender (Male, %)	91 (34.5)	87 (32.0)	178 (33.2)
Age (years)	45 ± 13	44 ± 13	44 ± 13
BMI	22.7 ± 3.6	22.8 ± 3.7	22.7 ± 3.7
Ethnicity (%)			
Han nationality	240 (90.9)	241 (88.6)	481 (89.7)
Minority nationality	24 (9.1)	31 (11.4)	55 (10.3)
Education level (%)			
University and above	66 (25.0)	90 (33.1)	156 (29.1)
High school	56 (21.2)	49 (18.0)	105 (19.6)
Junior high school	71 (26.9)	62 (22.8)	133 (24.8)
Primary school and below	43 (16.3)	48 (17.7)	91 (17.0)
Unknown	28 (10.6)	23 (8.5)	51 (9.5)
Residence (%)			
Urban area	156 (59.1)	164 (60.3)	320 (59.7)
Rural area	95 (36.0)	93 (34.2)	188 (35.1)
Unknown	13 (5.0)	15 (5.5)	28 (5.2)
Comorbidities (%)			
Diabetes	11 (4.2)	14 (5.1)	25 (4.7)
Malignant tumor	2 (0.8)	0	2 (0.4)
Silicosis	1 (0.4)	0	1 (0.2)
Diagnosis of immune disease (%)			
Systemic lupus erythematosus	79 (29.9)	84 (30.9)	163 (30.4)
Takayasu arteritis	17 (6.4)	17 (6.3)	34 (6.3)
Behcet's syndrome	50 (18.9)	54 (19.9)	104 (19.4)
Sjögren's syndrome	28 (10.6)	32 (11.8)	60 (11.2)
Rheumatoid arthritis	58 (22.0)	52 (19.1)	110 (20.5)
Ankylosing spondylitis	32 (12.1)	33 (12.1)	65 (12.1)
Newly diagnosed rheumatic diseases	64 (25.7)	67 (25.8)	131 (25.7)
Duration of rheumatic diseases (months)	48 [16–119]	54 [21–112]	53 [18–118]
Glucocoticoid use (in the past 2 years)^a^	172 (65.2)	164 (60.3)	336 (62.7)
≥50 mg/day	58 (22.0)	62 (22.8)	120 (22.4)
Course (weeks, [median, IQR])	2 [1–4]	2 [1–4]	2 [1–4]
30–49 mg/day	75 (28.4)	61 (22.4)	136 (25.4)
Course (weeks, [median, IQR])	2 [1–8]	5 [2–8]	4 [2–8]
15–29 mg/day	70 (26.5)	71 (26.1)	141 (26.3)
Course (weeks, [median, IQR])	6 [2–12]	6 [2–12]	6 [2–12]
<15 mg/day	79 (29.9)	82 (30.1)	161 (30.0)
Course (weeks, [median, IQR])	40 [8–95]	61 [23–95]	52 [12–95]
Maximum dose (mg/d)	37 [20–50]	40 [15–50]	40 [20–50]
Immunosuppressant use (in the past 2 years)	162 (61.4)	163 (59.9)	325 (60.6)
Cyclophosphamide	33 (12.5)	35 (12.9)	68 (12.7)
Methotrexate	58 (22.0)	54 (20.0)	112 (20.9)
Mycophenolate Mofetil	40 (15.2)	37 (13.6)	77 (14.4)
Cyclosporine A	10 (3.8)	16 (5.9)	26 (4.9)
Azathioprine	6 (2.3)	3 (1.1)	9 (1.7)
Tacrolimus	9 (3.4)	8 (2.9)	17 (3.2)
Leflunomide	31 (11.7)	34 (12.5)	65 (12.1)
Iguratimod	17 (6.4)	19 (7.0)	36 (6.7)
Tripterygium Glycosides	24 (9.1)	23 (8.5)	47 (8.8)
Biological agents (in the past 2 years)	18 (6.8)	23 (8.5)	41 (7.6)
Adalimumab	1 (0.4)	2 (0.7)	3 (0.6)
Infliximab	0	0	0
Etanercept	8 (3.0)	13 (4.8)	21 (3.9)
Golimumab	0	0	0
Tocilizumab	1 (0.4)	3 (1.1)	4 (0.7)
Rituximab	4 (1.5)	1 (0.4)	5 (0.9)
Tofacitinib	5 (1.9)	4 (1.5)	9 (1.8)
Evidence of previous tuberculosis	42 (15.9)	45 (16.5)	87 (16.2)
History of previous tuberculosis	26 (9.8)	24 (8.8)	50 (9.3)
Inactive tuberculosis lesions on chest imaging	32 (12.1)	29 (10.7)	61 (11.4)
Close contact with pulmonary tuberculosis	7 (2.7)	4 (1.5)	11 (2.1)
Laboratory tests [median, IQR]			
WBC (10e9/L)	6.95 [5.08–8.90]	6.81 [5.18–9.13]	6.90 [5.16–9.06]
N (%)	66.2 [57.7–74.9]	64.6 [57.2–73.3]	65.5 [57.6–74.1]
L (%)	23.5 [16.2–31.4]	24.7 [16.8–30.7]	24.0 [16.5–31.0]
Hb (g/L)	123 [109–139]	125 [113–137]	124 [111–138]
PLT (10e9/L)	243 [187–298]	244 [191–309]	243 [190–304]
ALT (U/L)	17 [12–26]	16 [11–25]	16 [11–25]
AST (U/L)	18 [13–22]	18 [14–23]	18 [14–23]
TBIL (μmol/L)	8.5 [6.2–11.7]	8.8 [6.1–12.0]	8.6 [6.1–11.8]
DBIL (μmol/L)	2.9 [2.0–4.3]	2.9 [2.1–4.3]	2.9 [2.1–4.3]
TP (g/L)	68 [62–73]	69 [63–75]	68 [62–73]
ALB (g/L)	38 [34–42]	39 [35–43]	38 [35–42]
ALP (U/L)	68 [52–86]	65 [49–86]	67 [51–86]
GGT (U/L)	24 [16–40]	22 [14–35]	23 [15–38]
Cr (μmol/L)	58 [51–72]	58 [51–70]	58 [51–71]
CRP (mg/L)	4.31 [1.43–16.98]	4.07 [1.14–13.80]	4.10 [1.27–15.15]
ESR (mm/h)	23 [11–47]	23 [8–42]	23 [10–45]
Fasting blood glucose (mmol/L).	4.7 [4.2–5.3]	4.8 [4.3–5.3]	4.8 [4.3–5.3]
T-SPOT.TB (SFCs/million PBMC)			
ESAT-6	88 [40–200]	100 [48–296]	96 [40–260]
CFP-10	72 [28–200]	86 [28–224]	80 [28–206]

BMI, Body Mass Index; IQR, Interquartile range; WBC, White Blood Cell; N, Neutrophil; L, Lymphocyte; Hb, Hemoglobin; PLT, Platelet; ALT, Alanine Aminotransferase; AST, Aspartate Aminotransferase; TBIL, Total Bilirubin; DBIL, Direct Bilirubin; TP, Total Protein; ALB, Albumin; ALP, Alkaline Phosphatase; GGT, Gamma–Glutamyl Transferase; Cr, Creatinine; Urea, Urea Nitrogen; CRP, C–reactive Protein; ESR, Erythrocyte Sedimentation Rate; SFC, Spot Forming Cell; PBMC, Peripheral Blood Mononuclear Cell; ESAT-6, 6kd Early Secreted Antigenic Target; CFP-10, 10kd Culture Filtrate Protein.

a Glucocorticoid categories reflect prednisone-equivalent exposure in the past 2 years; eligibility required ≥1 medication-related risk criterion (prednisone-equivalent ≥15 mg/day, immunosuppressant use, or planned TNFi initiation).

**Fig. 2:**
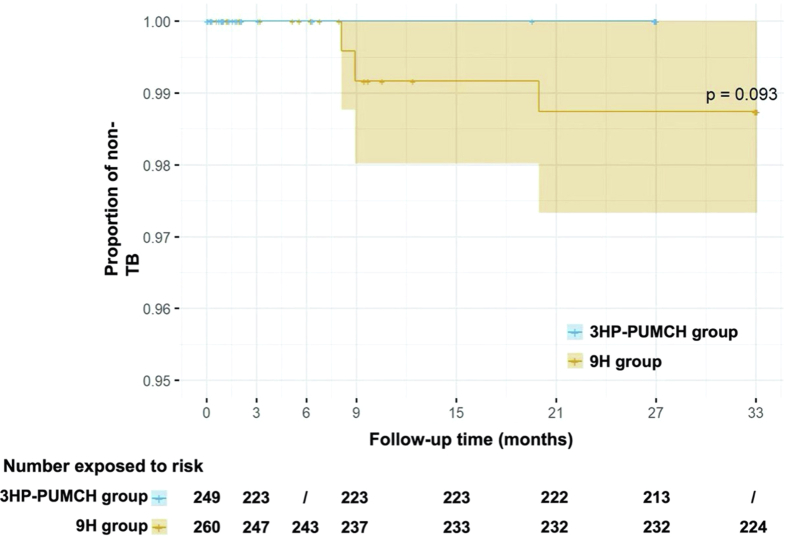
The occurrence of the primary endpoint depicted by the Kaplan–Meier curve.

No tuberculosis occurred among the 249 patients with rheumatic diseases in the 3HP-PUMCH group, with a cumulative incidence rate of 0.00% (0/249, 95% CI 0.00–1.47%). Three cases of tuberculosis occurred among the 260 patients with rheumatic disease in the 9H group, with a cumulative incidence rate of 1.15% (3/260, 95% CI 0.24%–3.34%). The difference in cumulative incidence rates between the two groups was −1.15 percentage points (95% CI −2.45 to 0.14). The upper limit of the CI (0.14 percentage points) was less than the prespecified non-inferiority boundary value of the rate difference (1.4 percentage points), achieving a non-inferiority result. However, the upper limit of the CI for the cumulative incidence rate difference was greater than 0.0 percentage points, a superior result was not further achieved. The result of the sensitivity analysis was consistent with those of the primary analyses (Table 2). The log-rank test yielded a p value of 0.093. However, given the limited number of primary events (n = 3), this result should be interpreted with caution (Fig. 2).

**Table 2:** Incidence of tuberculosis disease in patients with rheumatic diseases.

	3HP-PUMCH regimen	9H regimen	Difference in percentage points (95% CI)
**Modified intention-to-treat analysis**			
No. of events/total	0/249	3/260	
Cumulative rate % (95% CI)	0 (0–1.47)	1.15 (0.24–3.34)	−1.15 (−2.45 to 0.14)
No. of events/pys	0/504	3/650	
Incidence rate/100 pys (95% CI)	0 (0–0.73)	0.46 (0.10–1.35)	−0.46 (−0.98 to 0.06)
**Per-protocol analysis**			
No. of events/total	0/223	2/237	
Cumulative rate % (95% CI)	0 (0–1.64)	0.84 (0.10–3.02)	−0.84 (−2.01 to 0.32)
No. of events/pys	0/501	2/643	
Incidence rate/100 pys (95% CI)	0 (0–0.74)	0.31 (0.04–1.12)	−0.31 (−0.74 to 0.12)
**Intention-to-treat analysis**^a^			
No. of events/total	0/259	3/267	
Cumulative rate % (95% CI)	0 (0–1.41)	1.12 (0.23–3.25)	−1.12 (−2.38 to 0.13)
No. of events/pys	0/524	3/668	
Incidence rate/100 pys (95% CI)	0 (0–0.70)	0.45 (0.09–1.31)	−0.45 (−0.95 to 0.06)

H, Isoniazid; P, Rifapentine; PUMCH, Peking Union Medical College Hospital.

a The intention-to-treat analysis includes the 17 patients who declined treatment and were not included in the modified intention-to-treat population.

All three patients with endpoint events had SLE. They developed tuberculosis within 2 years of receiving TPT. Two cases were microbiologically confirmed: one patient developed disseminated tuberculosis, which was confirmed by next-generation sequencing of blood and ascitic fluid 1 month after completing the 9H regimen, while the other patient developed pulmonary tuberculosis with a positive sputum culture 12 months after completing TPT. The third patient was clinically diagnosed with lumbar tuberculosis and experienced a tuberculosis breakthrough 8 months after initiating the 9H regimen.

Permanent drug discontinuation rates as a result of serious adverse events or occurrence of tuberculosis were 2.8% (7/249) in the 3HP-PUMCH group and 1.9% (5/260) in the 9H group (p = 0.51). The proportions of patients with any adverse events were 13.7% (34/249) in the 3HP-PUMCH group and 18.8% (49/260) in the 9H groups (p = 0.11). The proportion of patients with grade 1 adverse events in the 9H group was higher than that in the 3HP-PUMCH group (11.5% [30/260] vs. 5.2% [13/249], p = 0.010). None of the deaths were attributed to preventive drugs (Table 3).

**Table 3:** Adverse events in patients with rheumatic diseases.

Outcome	3HP-PUMCH group (n = 249)	9H group (n = 260)	p value
Permanent drug discontinuation -no./total no. (%)			
For any reason	26 (10.4)	23 (8.8)	0.54
For any serious adverse event/occurrence of tuberculosis^a^	7 (2.8)	5 (1.9)^a^	0.51
For adverse drug reaction	13 (5.2)	10 (3.8)	0.46
Death-no./total no. (%)	2 (0.8)	4 (1.5)	0.72
Any adverse event-no. (%)	34 (13.7)	49 (18.8)	0.11
1 Adverse event	33 (13.3)	44 (16.9)	0.25
>1 Adverse event	1 (0.4)	5 (1.9)	0.24
Attribution -no. (%)			
Related to drug	24 (9.6)	39 (15.0)	0.066
Hepatotoxicity	11 (4.4)	27 (10.4)	0.010
Peripheral neuritis	3 (1.2)	2 (0.8)	0.96
Gastrointestinal reaction	3 (1.2)	1 (0.4)	0.59
Rash/hypersensitivity	4 (1.6)	4 (1.5)	1
Kidney damage	0	2 (0.8)	0.50
Leukopenia	2 (0.8)	4 (1.5)	0.72
Optic neuritis	0	0	/
Others	2 (0.8)	0	0.24
Not related to drug	9 (3.6)	9 (3.5)	0.93
Severity of adverse event-no. (%)			
Grade 1	13 (5.2)	30 (11.5)	0.010
Grade 2	7 (2.8)	7 (2.7)	0.94
Grade ≥3 (severe or higher)	4 (1.6)	2 (0.8)	0.38

H, Isoniazid; P, Rifapentine; PUMCH, Peking Union Medical College Hospital.

a One patient developed breakthrough tuberculosis during preventive treatment, which led to treatment discontinuation; this event was not classified as an adverse event.

The proportion of patients with adverse events related to preventive drugs was 9.6% (24/249) in the 3HP-PUMCH group and 15.0% (39/260) in the 9H group (p = 0.066), Although the proportion of drug-related adverse events was numerically lower in the 3HP-PUMCH group, this difference did not reach statistical significance. The proportions of permanent drug discontinuation resulting from adverse drug reactions were 5.2% (13/249) and 3.8% (10/260), respectively (p = 0.46). Hepatotoxicity was the most common adverse drug reaction, with a proportion of 10.4% (27/260) in the 9H group and 4.4% (11/249) in the 3HP-PUMCH group (p = 0.010). 81.5% (22/27) of the hepatotoxicity in the 9H group occurred within 3 months after the initiation of TPT (Table 3).

89.6% (223/249) of patients in the 3HP-PUMCH group and 91.2% (237/260) in the 9H group completed TPT (p = 0.54).

## Discussion

To our knowledge, this is the first RCT to evaluate the efficacy and safety of a short-course TPT regimen for LTBI in Chinese patients with high-risk rheumatic diseases. These patients face a significantly elevated risk of developing tuberculosis.

Building on our previous multicenter cross-sectional study on the prevalence and risk factors of tuberculosis in these patients, we selected patients with six specific types of common or high-risk rheumatic diseases who were also receiving glucocorticoids or immunosuppressive agents or were planning to initiate TNF inhibitor therapy. These participants were most susceptible to tuberculosis and most likely to benefit from TPT. Additionally, our 2-year follow-up results suggest that the 3HP-PUMCH regimen effectively reduced the risk of LTBI reactivation, with an efficacy non-inferior to that of the classic 9H regimen.

TPT of LTBI can be used to reduce the risk of developing tuberculosis by 60–90%.^22^ A network meta-analysis indicated that both the 9H and 3HP regimens significantly reduced the risk of tuberculosis (odds ratio [OR] 0.46 [95% CI 0.22–0.95] vs 0.36 [95% CI 0.18–0.73]).^23^ However, in another network meta-analysis, it was found that although the 3HP regimen was effective (RR 0.35, 95% CI 0.10–0.88), the 9H regimen did not significantly decrease the risk of developing tuberculosis (RR 0.49, 95% CI 0.07–1.59).^24^ The evidence supporting the efficacy and safety of TPT primarily comes from studies on patients with HIV, silicosis, or close contact with patients with tuberculosis. However, these populations differ significantly from patients with rheumatic diseases in clinical characteristics and immune status. Inherent immune disorders and the use of immunosuppressive therapies both place these patients at high risk of developing tuberculosis.^25^ WHO recommends extending the TPT to other high-risk populations as a crucial measure for tuberculosis control.^26^ However, patients with rheumatic diseases should be prioritized. The effectiveness of current TPT regimens in preventing LTBI reactivation in patients with rheumatic diseases under dual-risk still lacks direct, high-quality evidence. In this study, this concern was addressed by demonstrating that the 3HP-PUMCH regimen offers good protective effects in these high-risk patients during the 2-year follow-up period.

While the 3HP-PUMCH regimen demonstrated non-inferiority, it is worth noting that all three incident tuberculosis cases occurred in the 9H group, specifically among patients with SLE. SLE, one of the most prevalent rheumatic diseases, is characterized by a complex pathogenesis, heterogeneous clinical manifestations, and extensive multi-organ involvement,^27^ making LTBI screening and management particularly challenging.^17^^,^^18^^,^^28^ Although our study was not powered for superiority or formal subgroup comparisons, these observations suggest that patients with SLE may represent a particularly high-risk group requiring optimized TPT strategies. Further large-scale prospective studies are needed to confirm whether 3HP-PUMCH offers a distinct advantage over 9H in the SLE population. A 2007 epidemiological survey in China^29^ reported an isoniazid resistance rate of 16% in treatment-naive and 38.5% in treatment-experienced patients with positive acid-fast staining of sputum. N-acetyltransferase type 2 is crucial for isoniazid metabolism. In previous literature, it was reported that nearly 50% of the Asian population carries a rapid acetylator genotype, leading to inadequate exposure to isoniazid. By contrast, only 5% of Caucasians carried this genotype. This difference may partially explain the suboptimal protective effect of isoniazid monotherapy in Chinese patients with rheumatic diseases. Our team's meticulously conducted meta-analysis in patients with rheumatic diseases demonstrated that prolonged isoniazid monotherapy duration may enhance the effectiveness of TPT.^19^ Furthermore, a previous meta-analysis suggested that extending treatment to 12 months or longer could more effectively prevent LTBI reactivation, providing additional evidence-based support for optimizing TPT strategies.^24^ Therefore, an extended course of isoniazid monotherapy may be an alternative regimen for rifapentine-intolerant patients.

Regardless of TPT's effectiveness in preventing tuberculosis, its adverse drug reactions, including hepatotoxicity, peripheral nephropathy, rash, gastrointestinal discomfort, and influenza-like syndrome, cannot be overlooked.^26^ In Chinese patients with silicosis receiving the standard 3HP regimen (weekly administration), the proportion of adverse events was as high as 70.4%, with 28.3% of patients discontinuing the drugs.^12^ Additionally, Gao et al. reported an unexpectedly high frequency of hepatotoxicity in people between the ages of 50 and 69 years following the 3HP regimen in China. This led to the premature cessation of the trial and a reduction in treatment duration from 3 months to 8 weeks.^13^

Genetic background significantly influences the risk of adverse drug reactions to anti-tuberculosis drugs. A pharmacokinetic study of the standard 3HP regimen for TPT in Chinese patients with silicosis revealed a fast metabolism of isoniazid and a slow metabolism of rifapentine. This potentially contributes to the poor tolerance to the standard 3HP regimen.^14^ Consequently, we adjusted the regimen to 3 months of daily isoniazid and twice-weekly rifapentine to improve patient tolerance by increasing the number of doses while reducing the amount per dose. Our findings showed a favorable and clinically meaningful safety profile for 3HP-PUMCH compared with the 9H regimen. The rate of hepatotoxicity in the 3HP-PUMCH group was lower than that in the 9H group, which is consistent with previous reports.^23^ Longer treatment duration may contribute to a higher risk of hepatotoxicity. However, in our study, 22 patients (8.5%, 22/260) in the 9H group had hepatotoxicity within the first 3 months, significantly higher than the 4.4% (11/249) in the 3HP-PUMCH group. This unexpected finding requires further investigation. Although our study did not include dedicated pharmacokinetic or pharmacogenomic substudies to directly confirm the metabolic hypothesis for rifapentine in the rheumatic diseases population, the observed clinical outcome—a significantly reduced hepatotoxicity rate in the 3HP-PUMCH regimen—serves as robust clinical evidence supporting the success of the dose-frequency modification in mitigating potential rifapentine-related accumulation toxicity in this vulnerable group.

Completion rates are also important factors in selecting a TPT regimen. A systematic review and meta-analysis revealed that shorter regimens can improve completion rates. Compared with the 12-month placebo group, patients receiving 3–4 months regimens including rifapentine achieved a higher completion rate (OR 3.58, 95% CI 1.40–8.83), while no significant difference was observed with the 9H regimen (OR 1.64, 95% CI 0.57–4.45).^24^ Another meta-analysis also showed a higher completion rate in patients receiving 3HP than in those receiving 6H or 9H (RR 1.3, 95% CI 1.24–1.37),^7^ However, in our study, the completion rate was approximately 90% in both groups, which was different from the results of studies in other populations. We hypothesized that rheumatic diseases are often lifelong and require long-term medication and follow-up. However, patients with rheumatic diseases have a much higher acceptance of the length of TPT courses than other populations. Our survey of rheumatic disease patients’ preferences for TPT,^30^ indicated that more than half of the patients could tolerate a treatment duration of 9 months or even longer, which may reasonably explain the comparable completion rates observed in both groups. Nevertheless, patients with rheumatic diseases still prioritize regimens with fewer adverse reactions and shorter duration, reflecting their ongoing core requirements for TPT.

Other factors to consider when choosing a TPT regimen for high-risk populations include cost, duration of treatment, pill burden, and drug interactions. A WHO expert group of scientists, physicians, tuberculosis project managers, public health experts, sponsors, and civil society representatives identified nine priority elements in the selection of TPT regimens, among which efficacy, course of treatment, and safety ranked among the top three.^31^ A shorter treatment duration should not result in reduced efficacy or safety. Our study showed that the 3HP-PUMCH regimen was comparably effective in preventing LTBI reactivation, with less hepatotoxicity and shorter treatment duration, making it a superior choice to the 9H regimen for patients with rheumatic diseases.

Our study has some limitations. First, the pharmacokinetic and pharmacogenomic data were not collected, so our mechanistic interpretation of the 3HP-PUMCH regimen remains indirect. Second, although this was a multicenter randomized trial, the sample size and low number of events limited subgroup analyses by specific rheumatic disease type or concomitant therapies, or individual research centers. Third, comorbidities and concomitant medications were not comprehensively captured beyond diabetes mellitus, malignancy, and silicosis; therefore, we could not assess whether other comorbidities (e.g., hypertension or renal insufficiency) and related medications influenced adverse events. Fourth, due to the anticipated low incidence rate of the primary endpoint over the planned follow-up period under TPT and constraints in clinical feasibility and resources, we prespecified an expected risk difference of −2.5 percentage points (rather than the conventional 0 percentage points) for the sample size calculation, informed by prior evidence in comparable high-risk populations. We acknowledge that assuming a negative risk difference can inflate nominal power and that the trial was powered for non-inferiority, not superiority; superiority analyses are therefore exploratory. Nonetheless, the prespecified non-inferiority margin was met, supporting the non-inferiority conclusion. In addition, the observed cumulative incidence in the control group (1.15%) was lower than the projected rate (3.6%), resulting in only three tuberculosis events overall and limited precision around the primary estimate. Therefore, while the prespecified non-inferiority margin was met, the low event rate reduces certainty and limits conclusions beyond non-inferiority. Fifth, the COVID-19 pandemic overlapped with our study's follow-up period. While pandemic-related social distancing and healthcare disruptions may have influenced tuberculosis exposure and reporting, we implemented mitigation measures, such as telephone follow-up and continued independent endpoint adjudication, to ensure the reliability and integrity of the data. Finally, the efficacy was evaluated based on a 2-year follow-up period. The long-term efficacy of the 3HP-PUMCH regimen in preventing LTBI reactivation remains unclear and requires extended follow-up. Additionally, while our study included nine centers across diverse geographic regions in China, only patients with high-risk rheumatic diseases were enrolled. This cohort is representative of the patients prioritized for TPT in clinical practice, our findings do not sufficiently reflect the condition of the general rheumatic disease population or those managed in primary care settings. Future RCTs with larger sample sizes and long-term follow-up are needed to validate these findings and explore these potential subgroup differences.

In conclusion, in this multicentre, non-inferiority trial among high-risk patients with rheumatic diseases, 3HP-PUMCH was non-inferior to 9H for preventing tuberculosis disease over 2 years, with high completion and an acceptable safety profile. Hepatotoxicity occurred less frequently with 3HP-PUMCH than with 9H in our study. 3HP-PUMCH may represent a reasonable TPT option for patients with rheumatic diseases in similar settings. Further studies in other populations and programmatic settings—particularly where standard 3HP is well tolerated—are needed to confirm generalizability.

## Contributors

Xiaoqing Liu, as the principal investigator, conceived and designed the study, secured funding, and supervised all aspects of the research. Lifan Zhang coordinated the study implementation, oversaw patient enrollment, performed the initial data analysis, drafted the manuscript, and revised subsequent versions. Hongbin Li and Ping Zhu provided critical methodological guidance, contributed to clinical evaluation and data interpretation, and substantially reviewed and revised the manuscript for intellectual content. Yujie He and Wenwen Wang contributed equally to the initial study design and execution, patient recruitment, and were involved in data interpretation. Lijun Wu, Xiaoxia Zuo, Sheng Chen, Yanping Zhao, Shengyun Liu, Huaxiang Wu, Cainan Luo, Yaou Zhou, Ran Wang, Juan Zhang, Chunlei Li, Kui Zhang, Wenjie Zheng, Lidan Zhao, Jinjing Liu, Wen Zhang, Baotong Zhou, Guiren Ruan, and Qian Wang were essential for patient recruitment, clinical data collection, and management across the participating centers. Mengtao Li, Yan Zhao, Xiaofeng Zeng, and Fengchun Zhang contributed significantly to the clinical evaluation of patients and the interpretation of the results. Yuelun Zhang conducted the primary statistical analyses. Lifan Zhang, Yujie He and Wenwen Wang verified the data. All authors substantially contributed to the manuscript, approved the final version, and agree to be accountable for all aspects of the work.

## Data sharing statement

Study protocol, statistical analysis plan and data will be available through email with publication after getting permission from corresponding author.

## Declaration of interests

We declare no competing interests.

## Appendix A Supplementary data

Supplementary Tables

Protocol
