# Supplementary material for: Efficacy, safety, and completion of modified short-course rifapentine and isoniazid for latent tuberculosis infection in patients with high-risk rheumatic disease: a multicentre, open-label, randomized, non-inferiority trial
Source: eClinicalMedicine. 2026 Mar 13;94:103831. doi: 10.1016/j.eclinm.2026.103831 (PMC13133530; doi:10.1016/j.eclinm.2026.103831)
Supplement: Supplementary Tables [file mmc1.docx]

| Supplementary Table 1. Characteristics and Independent Adjudication of 4 Participants with Pre-existing TB Identified Post-randomization | | | | | | | | | | | |
| --- | --- | --- | --- | --- | --- | --- | --- | --- | --- | --- | --- |
| ID | Gender | Age | Diagnosis of RD | Date of Randomization | Date of Discontinuation | Group | Protocol deviations | Whether TPT was initiated | TBD Diagnosis Date | TBD Diagnosis Method | EAC Adjudication Conclusion |
| 117 | Female | 38 | SLE | 2019/8/30 | 2019/9/6 | 3HP-PUMCH | Prevalent TBD | No | 2019/9/6 | Colonic mucosal biopsy (colonoscopy): histopathology consistent with TB, acid-fast staining positive | Prevalent intestinal TB identified post-randomization. Confirmed as pre-existing disease at baseline; ineligible per exclusion criterion 'a'. |
| 157 | Male | 50 | SLE | 2019/11/8 | 2020/4/20 | 9H | Prevalent TBD | Yes | 2020/4/20 | Xpert MTB/RIF positive (sputum) | Pulmonary TB (Xpert+) 5 months post-randomization. Independent committee's blinded retrospective review of baseline CT identified subtle active lesions missed at screening. Confirmed as prevalent TB at randomization. Not an incident event. Ineligible per exclusion criterion 'a'. |
| 294 | Female | 51 | SLE | 2020/6/19 | 2020/7/10 | 9H | Prevalent TBD | No | 2020/7/10 | Culture positive for MTB (Pleural fluid) | Prevalent tuberculous pleurisy identified post-randomization. Confirmed as pre-existing disease at baseline; ineligible per exclusion criterion 'a'. |
| 438 | Female | 64 | SLE | 2020/11/11 | 2020/11/25 | 3HP-PUMCH | Prevalent TBD | No | 2020/11/25 | Xpert MTB/RIF positive (BALF) | Prevalent pulmonary TB identified post-randomization. Confirmed as pre-existing disease at baseline; ineligible per exclusion criterion 'a'. |
| EAC: Endpoint Adjudication Committee, BALF: Bronchoalveolar Lavage Fluid | | | | | | | | | | | |

| Supplementary Table 2. Frequency and Severity of Adverse Events Stratified by CTCAE Grade. | | | | | | | | |
| --- | --- | --- | --- | --- | --- | --- | --- | --- |
|  | 3HP-PUMCH group (n=249) | | | | 9H group (n=260) | | | |
| Adverse Events (AEs) | All AEs n (%) | Related to TPT n (%) | Grade 1～2 n (%) | Grade ≥3 n (%) | All AEs n (%) | Related to TPT n (%) | Grade 1～2 n (%) | Grade ≥3 n (%) |
| Hepatotoxicity | 12(4.8) | 11(4.4) | 10(4.0) | 1(0.4) | 30(11.5) | 27(10.4) | 25(9.6) | 2(0.8) |
| Peripheral neuritis | 3(1.2) | 3(1.2) | 2(0.8) | 1(0.4) | 3(1.2) | 2(0.8) | 2(0.8) | 0 |
| Gastrointestinal reaction | 3(1.2) | 3(1.2) | 2(0.8) | 1(0.4) | 1(0.4) | 1(0.4) | 1(0.4) | 0 |
| Rash/hypersensitivity | 4(1.6) | 4(1.6) | 4(1.6) | 0 | 6(2.3) | 2(0.8) | 2(0.8) | 0 |
| Kidney damage | 0 | 0 | 0 | 0 | 2(0.8) | 2(0.8) | 2(0.8) | 0 |
| Leukopenia | 7(2.8) | 2(0.8) | 2(0.8) | 0 | 5(1.9) | 2(0.8) | 2(0.8) | 0 |
| Optic neuritis | 0 | 0 | 0 | 0 | 0 | 0 | 0 | 0 |
| Others | 4(1.6) | 2(0.8) | 1(0.4) | 1(0.4) | 3(1.2) | 0 | 0 | 0 |
| Permanent drug discontinuation | 26(10.4) | 13(5.2) | 9(3.6) | 4(1.6) | 23(8.8) | 10(3.8) | 8(3.1) | 2(0.8) |
| Death | 2(0.8) | 0 | 0 | 0 | 2(0.8) | 0 | 0 | 0 |

| Supplementary Table 3. Clinical Details of Deaths During the Study | | | | | | | | | |
| --- | --- | --- | --- | --- | --- | --- | --- | --- | --- |
| ID | Gender | Age(years) | Rheumatic Disease | TPT Group | Date of Randomization | Date of deah | Cause of death | TPT completed | Assessed as Related to TPT |
| 13 | Female | 54 | SLE | 9H | 2018/10/6 | 2019/9 | Unkown | Yes | No |
| 41 | Male | 45 | SLE | 9H | 2019/4/15 | 2019/10/2 | Unkown | No | No |
| 89 | Female | 44 | SLE | 3HP-PUMCH | 2019/7/9 | 2021/4/22 | Colon cancer | Yes | No |
| 135 | Male | 32 | SLE | 9H | 2019/9/27 | 2019/12/31 | Fungal meningoencephalitis | No | No |
| 178 | Female | 57 | SLE | 3HP-PUMCH | 2019/12/18 | 2020/2/9 | Aortic aneurysm rupture | No | No |
| 506 | Female | 26 | TA | 9H | 2021/3/26 | 2021/8/31 | Sudden cardiac death | No | No |
| SLE: Systemic lupus erythematosus, TA:Takayasu arteritis  All deaths were reviewed by the local clinicians and the study safety committee. None were considered related to TPT, based on timing, clinical course, and cause of death. For patients ID 13 and ID 41, specific causes of death were not disclosed by their families; however, no treatment-related AEs were recorded during their TPT period. | | | | | | | | | |
