# Supplementary material for: Efficacy, safety, and completion of modified short-course rifapentine and isoniazid for latent tuberculosis infection in patients with high-risk rheumatic disease: a multicentre, open-label, randomized, non-inferiority trial
Source: eClinicalMedicine. 2026 Mar 13;94:103831. doi: 10.1016/j.eclinm.2026.103831 (PMC13133530; doi:10.1016/j.eclinm.2026.103831)
Supplement: Protocol [file mmc2.docx]

**Efficacy, Safety, and Completion of Modified Short-Course Rifapentine and Isoniazid for Latent Tuberculosis Infection in** **High-Risk Rheumatic Disease Patients:**

**A Multicenter Randomized Controlled Trial**

**Administrative Information**

**Protocol Version** Version 2

**Protocol Date** April 17, 2020 (Revision Date)

**Trial Registration** Chinese Clinical Trial Registry (ChiCTR)

**Registration Number** ChiCTR1800018242

**Registration Date** September 6, 2018

**Funding Sources**

National Science and Technology Major Project (2017ZX10201302-003, 2014ZX10003003); National High Level Hospital Clinical Research Funding (2022-PUMCH-C-013); National Natural Science Foundation of China (82373648).

**Role of Funders**

The funding sources had no role in the study design; in the collection, analysis, and interpretation of data; in the writing of the report; or in the decision to submit the paper for publication.

**Principal Investigator** Xiaoqing Liu

**Contact for Correspondence** Xiaoqing Liu (E-Mail: liuxqpumch@vip.163.com)

**Index**

[1. Background 3](#_Toc213415097)

[2. Objectives 3](#_Toc213415098)

[3. Methods 3](#_Toc213415099)

**[3.1.](#_Toc213415100)****[Study design](#_Toc213415100)** [3](#_Toc213415100)

**[3.2.](#_Toc213415101)****[Study population](#_Toc213415101)** [3](#_Toc213415101)

**[3.3.](#_Toc213415102)****[Randomization](#_Toc213415102)** [4](#_Toc213415102)

**[3.4.](#_Toc213415103)****[Interventions](#_Toc213415103)** [4](#_Toc213415103)

**[3.5.](#_Toc213415104)****[Outcomes](#_Toc213415104)** [4](#_Toc213415104)

**[3.6.](#_Toc213415105)****[Data collection and follow up](#_Toc213415105)** [5](#_Toc213415105)

**[3.7.](#_Toc213415106)****[Statistical Considerations](#_Toc213415106)** [5](#_Toc213415106)

[4. Monitoring and Oversight 6](#_Toc213415107)

[5. Ethics and Dissemination 7](#_Toc213415108)

**[5.1.](#_Toc213415109)****[Research Ethics Approval](#_Toc213415109)** [7](#_Toc213415109)

**[5.2.](#_Toc213415110)****[Informed Consent](#_Toc213415110)** [7](#_Toc213415110)

**[5.3.](#_Toc213415111)****[Protocol Amendments](#_Toc213415111)** [7](#_Toc213415111)

**[5.4.](#_Toc213415112)****[Dissemination Policy](#_Toc213415112)** [7](#_Toc213415112)

1. **Background**

China is a high-burden country for tuberculosis (TB). Mathematical models suggest that even under the most optimistic diagnosis and treatment scenario, it will be difficult to achieve the current global TB control targets in China ^[1]^. Therefore, it is necessary to strengthen the management of latent tuberculosis infection (LTBI) and TB preventive treatment (TPT) for high-risk populations.

Patients with rheumatic diseases (RDs) are high-risk groups for TB infection and disease. Rheumatic diseases affect more than 20 million people in China., accounting for 20% of the global burden. Our study (epidemiological study and therapeutic evaluation of rheumatic patients with tuberculosis, ETHERTB) has found that the prevalence of active tuberculosis (ATB) among RDs patients is 882/100,000, significantly higher than that of the general population.

The LTBI population have a 5%-10% lifetime risk of progressing to ATB^[2, 3]^. The prevalence of LTBI nearly 20% in China ^[4]^. Due to the underlying disease and use of immunosuppressive medications, the risk of developing ATB in RDs patients is much higher than that of the general population. Therefore, conducting TPT among RDs patients with LTBI in China is of great significance in reducing the incidence and mortality of TB.

Nine-month isoniazid (INH) monotherapy (9H) is a standard TPT regimen recommended by the WHO. However, its effectiveness is limited by poor treatment complicance, partly owing to the long duration of the regimen. WHO recommended a 3-month regimen of weekly rifapentine (RFT) and INH (3HP) as an alternative ^[5]^. However, the safety profile of 3HP in Chinese population raises concerns ^[6]^.

Due to the high clinical heterogeneity, complex disease conditions and medications in RDs patents, the high-quality studies on TPT in this population is very limited. Considering the high risk of ADRs due to existed organ damage and concomitant medication which is almost inevitable in RDs patients, TPT regimens should be selected with caution. Based on previous studies and clinical experience from our center, we modified the 3HP regimen to 3HP-PUMCH, which involves twice weekly RFT at 450mg and daily INH at a maximum dose of 300mg for 3 months. We evaluated the efficacy, safety and completion of 3HP-PUMCH compared to the standard 9H regimen in high-risk RDs patients with LTBI.

1. **Objectives**
   1. To determine the non-inferiority of the 3HP-PUMCH regimen compared to the standard 9H regimen in preventing ATB in high-risk RDs patients with LTBI.
   2. To compare the safety profile and treatment completion rates between the two regimens.
2. **Methods**
   1. **Study design**

The study is a multi-center, open-label, non-inferiority, randomized controlled trial.

- 1. **Study population**
     1. Eligible criteria

1. Aged 18-70 years;
2. Diagnosed with at least one of the high-risk RDs including systemic lupus erythematosus (SLE), Takayasu's arteritis (TA), Behcet's disease (BD), primary Sjogren's syndrome (SS), rheumatoid arthritis (RA) and ankylosing spondylitis (AS);
3. Not currently using biologics;
4. Taking any of the following medications: ①≥15mg daily of prednisone or equivalent; ②at least one of the following immunosuppressive agents: methotrexate (MTX), cyclophosphamide (CTX), mycophenolate mofetil (MMF), cyclosporine A (CsA), azathioprine (AZA), tacrolimus (FK-506), leflunomide, iguratimod and tripterygium wilfordii; ③initiating TNF inhibitors treatment.
5. Positive for blood T-SPOT.TB (≥24 SFCs/10^6^ PBMC).
   - 1. Exclusion criteria:
6. Suspected or confirmed ATB,
7. Seropositive status for HIV-Ab,
8. Seropositive status for HCV-Ab or HBV surface antigen,
9. Severe liver damage (total bilirubin > 3mg/dL or aminotransferase > 2ULN) or liver cirrhosis,
10. Prior use of TPT,
11. History of allergy to INH or RFT,
12. Pregnancy or lactation.
    1. **Randomization**

Participants were randomly allocated in a 1:1 ratio into the 9H and 3HP-PUMCH groups using a computer-generated random sequence. Randomization was stratified in the research center. A random sequence was generated using a fixed-block randomization method with a block length of 4. Allocation concealment was maintained using a web-based central randomization system.

- 1. **Interventions**
     1. Experimental Group

3HP-PUMCH regimen: INH once daily, 5 mg/kg (with a maximum daily dose of 300 mg), combined with RFT twice a week, 450 mg each time, for 3 months.

- - 1. Control Group

9H regimen: INH once daily, 5 mg/kg (with a maximum daily dose of 300 mg), for 9 months.

- - 1. Concomitant Treatment

RDs Treatment: The patient's baseline RDs treatment was followed according to the best available clinical guidelines/consensus and adjusted as needed by the treating clinician. A uniform RD treatment regimen was not stipulated.

- 1. **Outcomes**
     1. **Primary outcomes**

The occurrence of ATB up to the end of the follow-up period (24 months after randomization).

1. Diagnosis criteria:

- Microbiologically confirmed ATB: Biological specimens were positive by smear microscopy, culture, Xpert MTB/RIF, or other molecular biological diagnostic methods.
- Clinically diagnosed ATB: A clinical diagnosis of ATB was made when patients present characteristic symptoms (e.g., fever, cough, chest pain, night sweats, weight loss), supported by highly suggestive laboratory and imaging findings. Additionally, the diagnosis must be corroborated by a positive response to empiric TB treatment.

1. Diagnosis Adjudication:

All suspected ATB cases were reviewed by an external Endpoint Event Adjudication Committee (comprising two TB and one rheumatology experts) who were blinded to the TPT regimen allocation.

- - 1. **Secondary outcomes**

1. Permanent discontinuation of TPT due to serious adverse events (SAEs) or ATB occurrence.
2. ADRs related to prophylactic drugs (assessed by WHO-UMC causality criteria).
3. Completion rate of TPT.

Completion of treatment are defined as the consumption of at least 22 doses of RFT and at least 81 doses of INH within 16 weeks for the 3HP-PUMCH group, and 243 doses of INH within 52 weeks for the 9H group.

- 1. **Data collection and follow up**
     1. **Data collection:**

Patient data will be collected by trained researchers using a standardized case report form (CRF). At baseline, demographic characteristics, diagnosis and course of RDs, current medication use (including glucocorticoids and/or immunosuppressants), and a series of laboratory tests will be conducted to assess participants' general health. These tests will include hematological parameters, liver and renal function, inflammatory markers, and fasting blood glucose.

- - 1. **Follow-up procedures:**

Participants in the 3HP-PUMCH group will attend outpatient follow-up visits at their recruitment center at months 1, 2, and 3 during preventive treatment. At each visit, clinical assessments and laboratory tests, including hematological parameters and liver and renal function tests, will be conducted. Medication adherence and adverse events will be documented, with particular attention to any drug-related side effects. After completing TPT, participants will return for follow-up visits at months 6, 12, 18, and 24 to assess the incidence of ATB.

Participants in the 9H group will attend outpatient follow-up visits at months 1, 2, 3, 6, and 9 during preventive treatment. The same set of clinical and laboratory assessments, as well as monitoring for medication adherence and adverse events, will be performed. Post-treatment visits will also be conducted at months 6, 12, 18, and 24 to record any incidence of ATB.

- - 1. **Medication adherence assessment**

Medication adherence will be evaluated using the 8-item Morisky Medication Adherence Scale (MMAS-8). The MMAS-8 is a self-reported questionnaire that categorizes adherence as high, medium, or low based on the total score. Assessments will be conducted at each follow-up visit during preventive treatment.

- 1. **Statistical Considerations**
     1. **Sample Size Calculation**

1. Baseline Risk Assumptions: The 2-year incidence of ATB in the 9H group is approximately 3.6% (annual incidence of 1.8%). This estimate is based on cohort studies conducted in high TB burden countries/regions^[7]^, with a focus on populations at high risk for TB, such as those receiving high-dose glucocorticoids or immunosuppressive treatment, or planning to receive iTNF. The study inclusion criteria clearly define this high-risk subgroup, rather than evaluating all patients with RDs in general. While no study has specifically included RD patients with these risk factors, the incidence estimate in this study is derived from available data in high-risk populations.
2. Non-Inferiority Margin (Δ): The pre-set margin is 1.4% (absolute risk difference). This margin is determined based on the clinically acceptable increase in risk (i.e., the maximum additional risk allowed for 3HP-PUMCH relative to 9H).
3. Expected Effect and Sample Size Limitation: The expected incidence in the 3HP-PUMCH group is 1.1% (with an expected risk difference of -2.5%). This hypothesis is informed by similar studies in the HIV population, which observed 1.01% vs 3.50% cumulative TB rates, yielding a risk difference of −2.49% in favour of 3HP^[8]^. While standard non-inferiority designs typically assume a 0% risk difference, this study incorporates this assumption based on the higher adherence and better protective effects of the short-course regimen containing rifapentine in clinical practice, as well as the expected reduction in toxicity with the modified regimen. This design reflects a practical clinical approach under conditions of limited resources and a lack of sufficient evidence. We acknowledge that this assumption might result in a sample size that may not meet statistical power requirements under a 0% risk difference assumption, which is one of the limitations of this study.
4. Statistical Power: 80% statistical power, one-sided alpha level of 0.025.
5. Sample Size Calculation: 236 participants per group.
6. Total Sample Size (Accounting for Attrition): Assuming a 10% attrition rate, a total of 526 eligible participants will be enrolled.
   - 1. **Analysis Sets**
7. Primary Analysis Set (mITT): The Modified Intention-to-Treat (mITT) analysis set includes all participants who were randomized and received at least one dose of the intervention drug.
8. Sensitivity Analysis Set (PP): The Per-Protocol (PP) analysis set includes all participants from the mITT set who completed the TPT.
   - 1. Statistical Methods
9. Primary Outcome Analysis

- The cumulative incidence and its 95% CI will be estimated using the Exact Method.
- Non-inferiority will be concluded by comparing the upper bound of the 95% CI for the difference in cumulative incidence with the pre-set non-inferiority margin (1.4%).
- Non-Inferiority Test Criteria: If the upper bound of the 95% CI is < 1.4%, non-inferiority will be considered demonstrated.
- Superiority Test: If non-inferiority is achieved, a further assessment will be made to determine whether the upper bound of the 95% CI is < 0.0% to test for superiority.

1. Secondary Outcome Analysis

Descriptive statistical methods will be used. The rates of adverse drug reactions (ADR), liver toxicity, and other outcomes will be compared using chi-square tests, and 95% CIs will be calculated.

1. Sensitivity Analysis

In the mITT population, incidence rates per person-year will be calculated, and the difference in incidence rates and its 95% CI will be estimated.

1. Software

Analysis will be conducted using R software (version 4.4.1).

1. **Monitoring and Oversight**

The trial is overseen by a Coordinating Center (Peking Union Medical College Hospital). An external Endpoint Event Adjudication Committee is established to independently review and validate all primary outcome events (ATB), ensuring objectivity and consistency.

1. **Ethics and Dissemination**
   1. **Research Ethics Approval**

This study received approval from the Ethics Committees of the participating hospitals (Approval Number: JS-1498). All procedures comply with the ethical standards of the institutional and national research committees and with the Helsinki declaration.

- 1. **Informed Consent**

All participants provided written informed consent prior to enrollment in the study.

- 1. **Protocol Amendments**

Any substantial changes to the protocol will be submitted to the Ethics Committees for approval and will be updated in the trial registry (ChiCTR).

- 1. **Dissemination Policy**

The results of this study will be published in peer-reviewed journals (such as this manuscript) and presented at scientific conferences. The final protocol will be accessible to readers as supporting information accompanying the main manuscript. All authors meet the criteria for authorship.

1. Lin HH, Wang L, Zhang H, Ruan Y, Chin DP, Dye C. Tuberculosis control in China: use of modelling to develop targets and policies. Bull World Health Organ 2015; 93 (11): 790-798. doi: 10.2471/BLT.15.154492.

2. Hartman-Adams H, Clark K, Juckett G. Update on latent tuberculosis infection. Am Fam Physician 2014; 89 (11): 889-896.

3. Houben RM, Dodd PJ. The Global Burden of Latent Tuberculosis Infection: A Re-estimation Using Mathematical Modelling. PLoS Med 2016; 13 (10): e1002152. doi: 10.1371/journal.pmed.1002152.

4. Gao L, Lu W, Bai L, Wang X, Xu J, Catanzaro A, et al. Latent tuberculosis infection in rural China: baseline results of a population-based, multicentre, prospective cohort study. Lancet Infect Dis 2015; 15 (3): 310-319. doi: 10.1016/S1473-3099(14)71085-0.

5. WHO. Guidelines on the management of latent tuberculosis infection. https://wwwwhoint/publications/i/item/9789241548908 2015.

6. Gao L, Zhang H, Xin H, Liu J, Pan S, Li X, et al. Short-course regimens of rifapentine plus isoniazid to treat latent tuberculosis infection in older Chinese patients: a randomised controlled study. Eur Respir J 2018; 52 (6): doi: 10.1183/13993003.01470-2018.

7. Zheng N. Analysis of Risk Factors for Reactivation of Latent Tuberculosis Infection and Evaluation of the Efficacy of Tuberculosis Preventive Treatment in Patients with Rheumatic Diseases 2018.

8. Sterling TR, Scott NA, Miro JM, Calvet G, La Rosa A, Infante R, et al. Three months of weekly rifapentine and isoniazid for treatment of Mycobacterium tuberculosis infection in HIV-coinfected persons. AIDS 2016; 30 (10): 1607-1615. doi: 10.1097/QAD.0000000000001098.
